# The Different Faces of the Selective Human Telomerase Inhibitor: BIBR1532 and Its Derivatives

**DOI:** 10.3390/cimb48090893

**Published:** 2026-09-01

**Authors:** Michał Kosno, Natalia Maciejewska, Kamila Wolska, Iwona Gabriel

**Affiliations:** 1 2nd Department of Radiology, Medical University of Gdańsk, Mariana Smoluchowskiego 17, 80-210 Gdańsk, Poland; michal.kosno@gumed.edu.pl; 2 Department of Pharmaceutical Technology and Biochemistry, Faculty of Chemistry, Gdańsk University of Technology, 11/12 Narutowicza Str., 80-233 Gdańsk, Poland; natalia.maciejewska@pg.edu.pl (N.M.); s189116@student.pg.edu.pl (K.W.)

**Keywords:** telomerase, telomere, BIBR1532, anticancer, docking studies

## Abstract

Telomerase is an interesting biological target for cancer treatment. Despite recent advances in telomerase research and the rapid development of new methodologies, significant challenges remain in identifying effective telomerase inhibitors with anticancer activity. In this manuscript, we provide computational studies to analyse how structural modification of BIBR1532, a selective and non-competitive telomerase inhibitor, may affect binding by a newly reported human telomerase model. BIBR1532 has been extensively studied in various cancer types, but analyses have shown its low anticancer activity. Further modification of BIBR1532 yielded compound BIBR1591 through a lipophilic morpholine ring extension of the benzoic acid. Interestingly, BIBR1591 showed approximately five-fold weaker inhibitory activity toward human telomerase relative to BIBR1532, but its in vivo preclinical studies have demonstrated significant anti-tumour and pro-apoptotic efficacy. Despite promising preclinical activities, neither compound has advanced to clinical trials, reportedly due to challenges with its pharmacokinetic properties. BIBR1532 suffers from poor solubility and low cellular uptake. This manuscript aims to provide an updated overview of human telomerase inhibition by BIBR1532 analogues and emphasise the importance of understanding its molecular mode of action based on the most updated literature. Using an in silico approach, we analysed how the structural modification of the BIBR scaffold influences the binding mode at the BIBR binding site recently discovered in the structure of human telomerase. We have also assessed whether reported analogues offer a measurable advantage over the parent compound.

## 1. Introduction

Telomerase remains an attractive, although difficult, target in anticancer drug discovery. Its relevance comes from the dependence of most tumour cells on telomere maintenance [1,2]. In differentiated somatic cells, telomeres shorten during successive rounds of DNA replication as a consequence of the end-replication problem, which prevents complete duplication of chromosome termini [3]. When this shortening reaches a critical point, cells usually lose further proliferative capacity and enter senescence, apoptosis, or replicative crisis [4]. Tumour cells evade this limitation mainly by restoring telomerase activity, which allows them to maintain telomere length and continue dividing [5].

**Figure 1:**
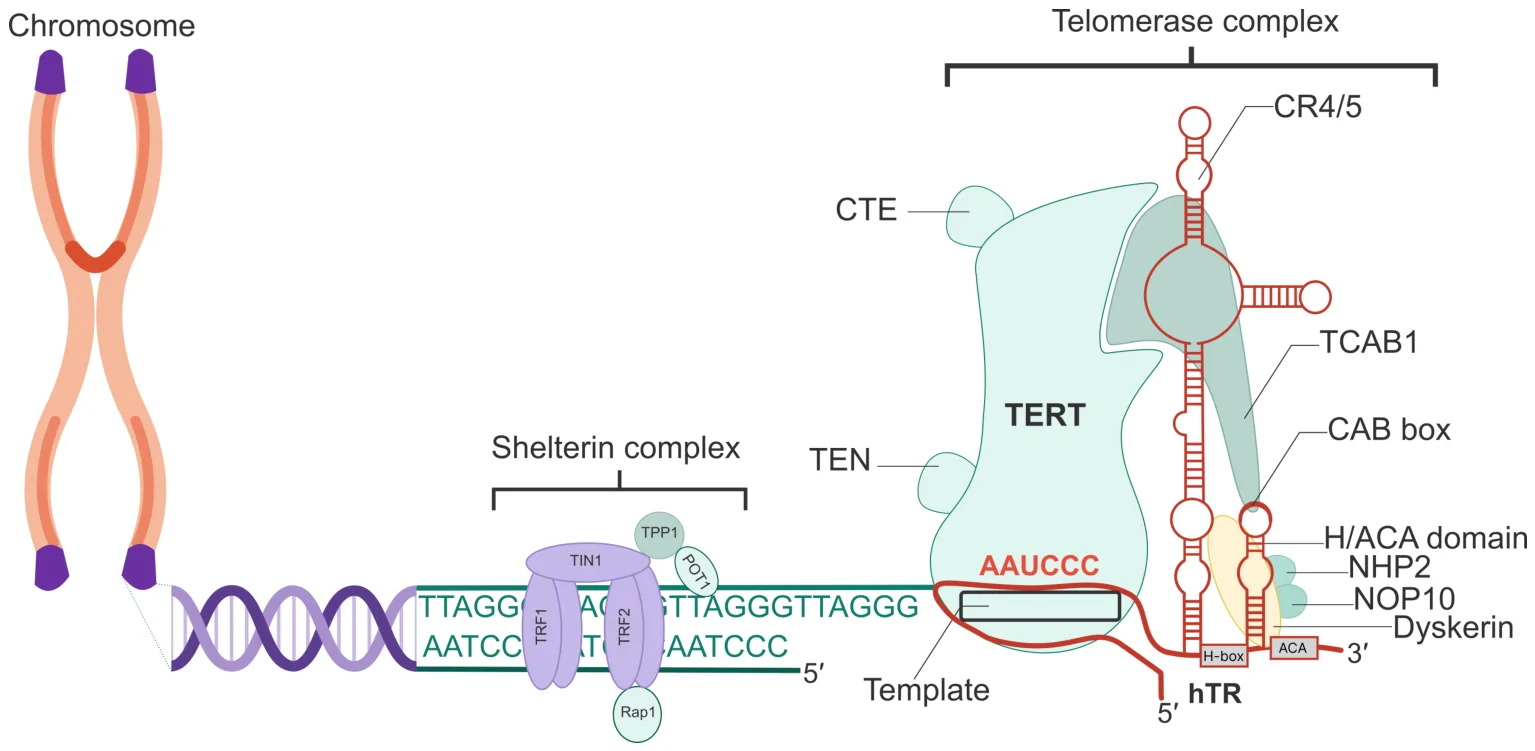
Schematic representation of the shelterin complex and human telomerase at chromosome ends. The telomerase holoenzyme is shown with the catalytic subunit hTERT, telomerase RNA (hTR), the CR4/5 domain, TCAB1, and the H/ACA ribonucleoprotein components. Adapted from [8].

Human telomerase is a ribonucleoprotein reverse transcriptase built around two core components: the catalytic subunit hTERT and the RNA template hTR/hTERC, which directs telomeric repeat synthesis (Figure 1) [6,7].

In cells, however, telomerase functions as a larger holoenzyme rather than as an isolated hTERT–hTR pair. Structural studies have shown that the human telomerase complex is organised into two major regions: a catalytic region and an H/ACA ribonucleoprotein module, with hTR helping to coordinate both parts of the enzyme [9]. Within the catalytic region, hTERT associates with conserved hTR elements, including the template/pseudoknot and CR4/5 domains, which help organise the active site and position the RNA–DNA substrate [9]. hTERT itself contains several major functional domains, including TEN, TRBD, RT and CTE, which cooperate during substrate binding, repeat synthesis and repeat-addition processivity [8,9]. The H/ACA module contains the H/ACA region of hTR associated with dyskerin, NOP10, NHP2 and GAR1, together with the Cajal body protein TCAB1 [8,9]. These proteins do not form the catalytic centre, but they are important for telomerase RNA stability, holoenzyme biogenesis, intracellular trafficking and proper telomerase function in cells [8,9].

Because telomerase activity is detected in most cancers but remains low or undetectable in many differentiated somatic tissues, selective telomerase inhibition has emerged as a plausible anticancer strategy [10,11]. Telomerase, however, is not a conventional cytotoxic target. Blocking the enzyme may not produce an immediate cellular response, because the consequences of telomere erosion usually appear only after several rounds of cell division [12].

**Figure 2:**
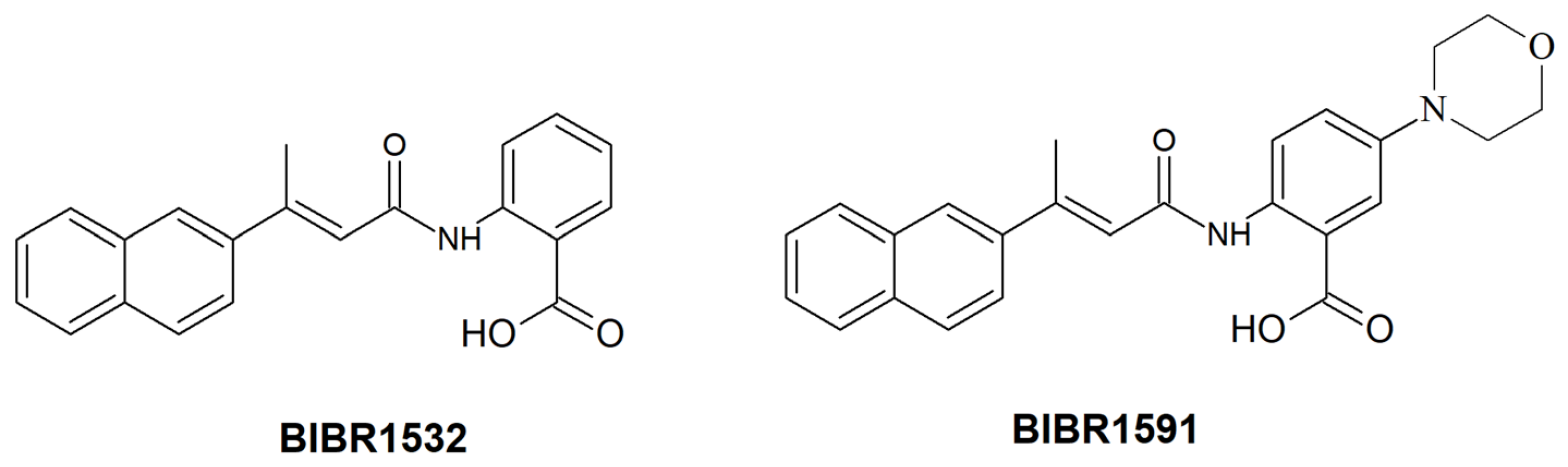
BIBR1532 and BIBR1591 structures.

BIBR1532 (2-[(E)-3-naphthalen-2-yl-but-2- enoylamino]-benzoic acid) is the best-established small-molecule reference compound in this field (Figure 2).

It is a synthetic non-nucleoside inhibitor of human telomerase and differs from agents that act mainly on telomeric DNA, such as G-quadruplex stabilizers or telomere-disrupting nucleoside analogues [13,14]. Rather than competing directly at the catalytic site, BIBR1532 acts allosterically and affects repeat addition processivity [15]. This mechanism, together with its selectivity and relatively simple scaffold, made the compound useful as a chemical probe and as a starting point for the design of related inhibitors [13,16,17,18,19,20,21]. Although BIBR1532 is valuable as a mechanistic probe, its properties are not sufficient for therapeutic development. Its apparent potency depends strongly on the experimental setting. Nanomolar inhibition has been reported in purified telomerase assays, while studies using crude cell extracts or cellular models generally show weaker activity [15,22]. This difference is relevant because biochemical inhibition does not necessarily translate into cellular activity or in vivo efficacy, which are additionally influenced by compound permeability, solubility, intracellular exposure, metabolic stability, and other pharmacokinetic factors. For BIBR1532, poor aqueous solubility, limited cellular uptake, and unfavourable pharmacokinetic behaviour are likely to reduce effective target engagement in biological systems [16,23]. As a result, BIBR1532 has remained an important reference compound but not a clinically developed telomerase inhibitor [12,24].

Subsequent work on BIBR1532 derivatives expanded the chemical space around the parent scaffold, but the reported activity data are methodologically heterogeneous. Across different studies, direct telomerase inhibition was assessed using purified or extract-based assays, RTQ-TRAP formats, single-point percentage inhibition measurements, and, in some cases, qPCR-based telomerase-related readouts [15,16,17,18,19,20,21]. As a result, the published potency values are not always directly comparable across series. Structural modification of the BIBR scaffold has produced analogues with weaker [17,19], comparable [16,21], or moderately improved telomerase inhibition [13,18], but clear and reproducible superiority over BIBR1532 itself has not been consistently demonstrated. These studies expanded the chemical space around the BIBR pharmacophore and provided useful structure–activity relationship data, but they also show that IC50 values must be interpreted in the context of the assay format, enzyme source, readout type, and experimental conditions. This distinction is particularly important for a comparative analysis of BIBR-derived inhibitors, because the values obtained in different assay systems cannot always be treated as equivalent measures of direct telomerase inhibition.

The structural interpretation of the BIBR scaffold interaction with telomerase has also changed. For several years, structure-guided design relied largely on the *Tribolium castaneum* TERT (tcTERT) model, which placed BIBR1532 in a C-terminal region and supported the proposal of a corresponding binding site in human TERT [25]. Since 2026, all reported novel BIBR1532-related analogues were designed on the BIBR pharmacophoric features defined using that model [16,17,18,19,20,21]. Bryan et al. provided an atomic view of telomerase in complex with inhibitor (PDB: 5CQG) and established that BIBR1532 binds to a conserved hydrophobic pocket (FVYL motif) on the outer surface of the tcTERT thumb domain [25]. The residues forming the tcTERT BIBR1532-binding pocket, F478, V491, Y551, and L554 of tcTERT corresponded to F1012, V1025, Y1089, and L1092 of human TERT (hTERT). The FVYL motif is located near TRBD residues that bind the activation domain (CR4/5) of hTR. Moreover, hTERT mutations of the FVYL pocket alter wild-type CR4/5 binding and cause telomere attrition in cells [25].

**Figure 3:**
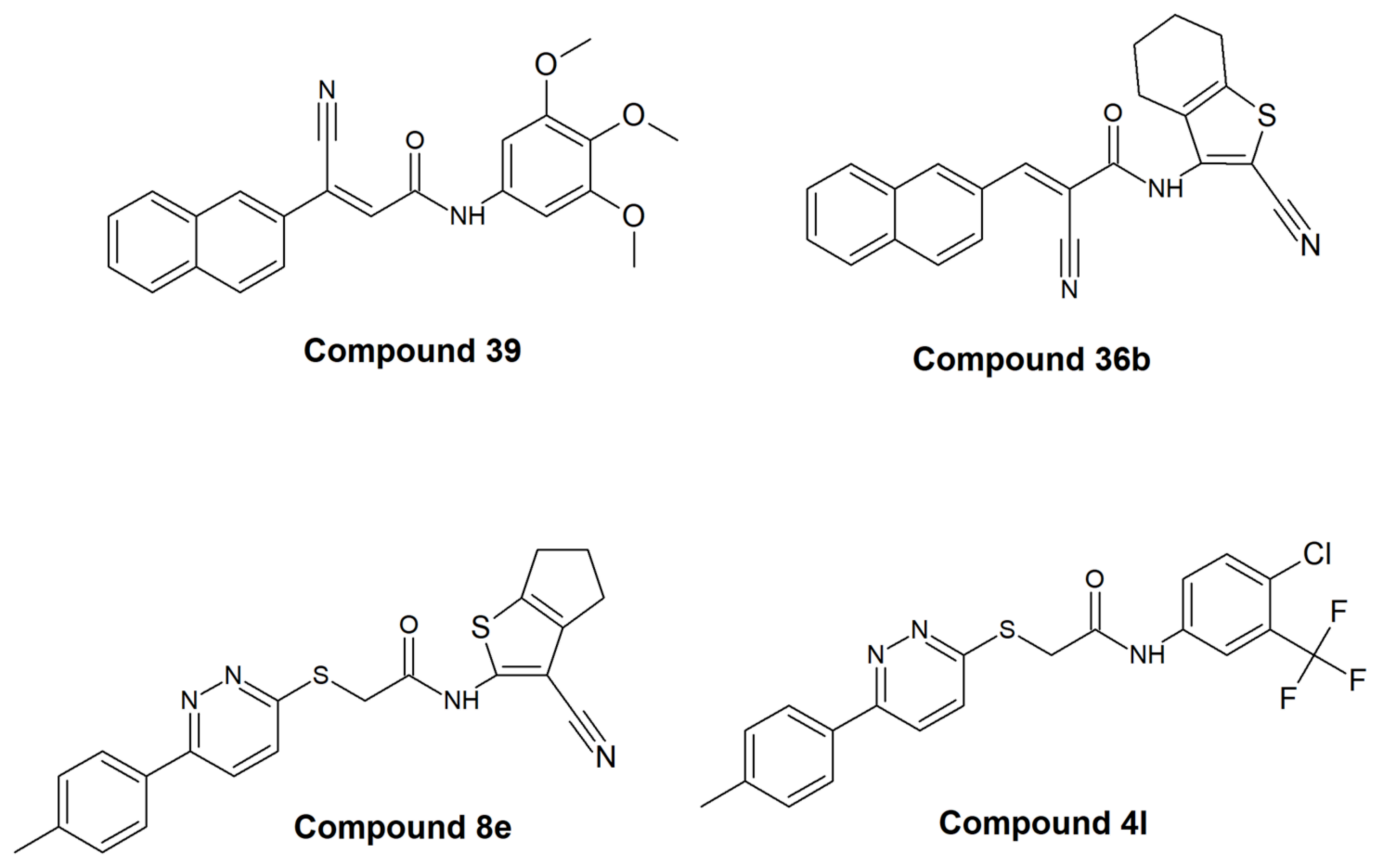
Telomerase inhibitors designed based on BIBR1532 docking mode in the tcTERT BIBR1532-binding pocket [16,17,19,21].

Molecular docking of BIBR1532 to the tcTERT BIBR1532-binding pocket showed that the naphthalene ring of the inhibitor (Figure 2) forms hydrophobic interactions with Leu554, Ile550, Phe494. The carboxyl group additionally interacts with Arg486 [21]. Further molecular docking studies performed for several BIBR-related analogues replicated this type of interaction [16,17,18,19,20,21]. Selected BIBR1532-related derivatives showing promising telomerase inhibitory activity and the BIBR1532-binding pocket reported for tcTERT are presented in Figure 3.

Comparison of the telomerase inhibitory activities of selected compounds is difficult because different assay formats were used. These included purified-enzyme or cell-extract-based assays. Nevertheless, for our studies we have chosen the most promising BIBR-related derivatives, published since 2020 [16,17,19,21]. The inhibitory activities of all compounds were compared with reference BIBR1532 with the use of the corresponding method (Table 1).

A TRAP-based assay identified compound **36b** ((2E)-2-Cyano-N-(3-cyano-4,5,6,7-tetrahydro-1-benzothiophen-2-yl)-3-(naphthalen-2-yl)prop-2-enamide) (Figure 3) as the most active derivative in this series. Its IC50 was 0.3 μM, compared with 0.2 μM for BIBR1532 [16]. A quantitative telomerase detection kit based on the real-time PCR assay identified compound **8e** (N-{3-Cyano-4H,5H,6H-cyclopenta[b]thiophen-2-yl}-2-{[6-(4-methylphenyl)pyridazin-3-yl]sulfanyl}acetamide) (Figure 3) as the most active derivative in this series. It inhibited telomerase activity of 58.36%, compared with 65.45% for BIBR1532 [17]. The inhibitory activity of the other candidate **4l** (N-(4-Chloro-3-(trifluoromethyl)phenyl)-2-((6-(p-tolyl)pyridazin-3-yl)thio)acetamide) (Figure 3) towards telomerase was assessed also using a Quantitative Telomerase Detection Kit [19]. Compound **4l** showed 64.95% telomerase inhibition compared to the BIBR1532 (72.74%) and BIBR1591 (68.75%) reference standards [20]. However, these values should not be compared directly across studies, as the assays differed in their detection systems, amplification procedures, enzyme sources, and experimental conditions. These methodological differences can substantially affect both the IC50 values and inhibition percentages. Therefore, the most meaningful comparison is between each tested compound and the corresponding reference inhibitor evaluated within the same study and under the same assay conditions. It should also be emphasised that these data reflect biochemical telomerase inhibition and should not be directly interpreted as evidence of cellular or antitumor activity. TRAP-PCR-ELISA was used by Liu et al. to evaluate the telomerase inhibitory activity of 64 compounds representing six structurally diverse series [21]. Compound **39** ((Z)-3-cyano-3-(naphthalen-2-yl)-N-(3,4,5-trimethoxyphenyl)acrylamide) (Figure 3) showed the strongest telomerase inhibitory potency, with an IC50 of 0.62 ± 0.12 μM. Under the same assay conditions, BIBR1532 showed an IC50 of 0.32 ± 0.07 μM [21].

Surprisingly, recently published cryo-EM structures of human telomerase (PDB: 9Q14, 9Q17, 9Q18, 9Q19 and 9Q0Z) in complex with BIBR1532 [26] do not support the binding mode described on the tcTERT model [25]. In the human enzyme, BIBR1532 was found in a distinct BIBR1532-binding pocket within the reverse transcriptase (RT) domain, located between the finger and palm regions of hTERT. The pocket lies close to the RNA template–DNA primer duplex, but the compound does not appear to block the catalytic centre directly [26]. Instead, structural and biochemical data indicate that BIBR1532 interferes with the movements required for template–DNA repositioning during repeat synthesis, particularly during the first rate-limiting nucleotide addition step [26]. This provides a firmer basis for understanding BIBR1532 selectivity and for reassessing earlier SAR assumptions derived from the *T. castaneum* TERT model.

This revised structural picture is particularly relevant for BIBR1591 (5-morpholin- 4-yl-2-[(E)-3-naphtalen-2-yl-but-2-enoylamino]-benzoic acid) (Figure 2) and newer derivatives [17,18]. BIBR1591 was developed as an elaborated analogue of BIBR1532, but it cannot be regarded simply as a more potent version of the parent compound [14]. Interestingly, BIBR1591 showed five times less inhibition toward hTERT (IC50 value of 0.47 μM), relative to BIBR1532 having an IC50 value of 0.093 μM.

Recent human telomerase structures suggest that some modifications introduced under earlier structural assumptions may not fit optimally within the recently identified human TERT BIBR1532-binding pocket [26,27]. These findings provide an important context for comparing reported telomerase IC50 values across BIBR1532, BIBR1591, and related derivatives.

In this manuscript, we delve into the BIBR1532 binding mode and analyse how the structural modification of the BIBR scaffold may affect binding to the recently identified BIBR1532-binding pocket in human TERT. By in silico methods we have also assessed whether the reported analogues offer a measurable advantage over the parent compound. Our computational results provide a possible structural explanation for the telomerase inhibitory activities reported previously in vitro (Table 1) [16,17,19,20,21].

## 2. Materials and Methods

### 2.1. Compounds Selection

Previous studies have identified several BIBR-related compounds with promising inhibitory activity. We decided to perform molecular docking analysis using selected compounds **36b** [16], **8e** [17], **4l** [19] and compound **39** [21] with the most promising inhibitory potential published since 2020.

### 2.2. Calculation of the Electrostatic Potential Surfaces and Docking Procedure

AutoDock Vina 1.1.2 [27,28] and Gnina 1.3 [29] were used to investigate the binding modes of four selected ligands: **39**, **8e**, **36b** and **4l** (Figure 3). We used three scoring functions for docking: Vina, Vinardo and GNINA. For each ligand and scoring method, nine docking poses were generated, resulting in a total of 27 poses per ligand. For each ligand, the combined set of poses obtained from Vina, Vinardo, and GNINA was subjected to clustering based on heavy-atom root-mean-square deviation (RMSD) using an RMSD cutoff of 2.0 Å. Clustering was performed with an in-house Python 3.14.5 script using RDKit library. From each resulting cluster, the pose with the best (lowest) scoring function value was selected as the cluster representative. The docking protocol was validated by redocking of the co-crystallised ligand BIBR1532 (PDB 9Q14) using AutoDock (Vina and Vinardo scoring function) and GNINA. The accuracy of the docking protocol was evaluated by redocking the co-crystallised BIBR1532 using all three scoring functions. The telomerase structures (PDB ID: 5CQG, 9Q14) [26] were retrieved from the Protein Data Bank and used as the docking receptors. For each ligand, the top-ranked pose with the lowest predicted binding energy was selected for further analysis. Electronic densities were computed at the wB97X-D4 def2-SVP level of theory [30]. Based on these results, electrostatic potential (ESP) maps were generated using ORCA 6.1.1 [31,32], mapped onto the van der Waals isosurface, and visualised using PyMOL (v3.1.8) [33].

## 3. Results

Recent advancements in cryo-electron microscopy and improved methods for human telomerase purification have substantially expanded our understanding of the enzyme. In particular, structures of telomerase in complex with BIBR1532 have provided new insight into its molecular mechanism of inhibition. To investigate the molecular mechanism of action of reported BIBR-related derivatives we performed docking analysis based on the most recent human TERT structural data (PDB ID 9Q14) [26]. The results were compared with those obtained using the previously published tcTERT model (PDB ID: 5CQG) [25].

### Molecular Docking and Electrostatic Potential Maps

**Figure 4:**
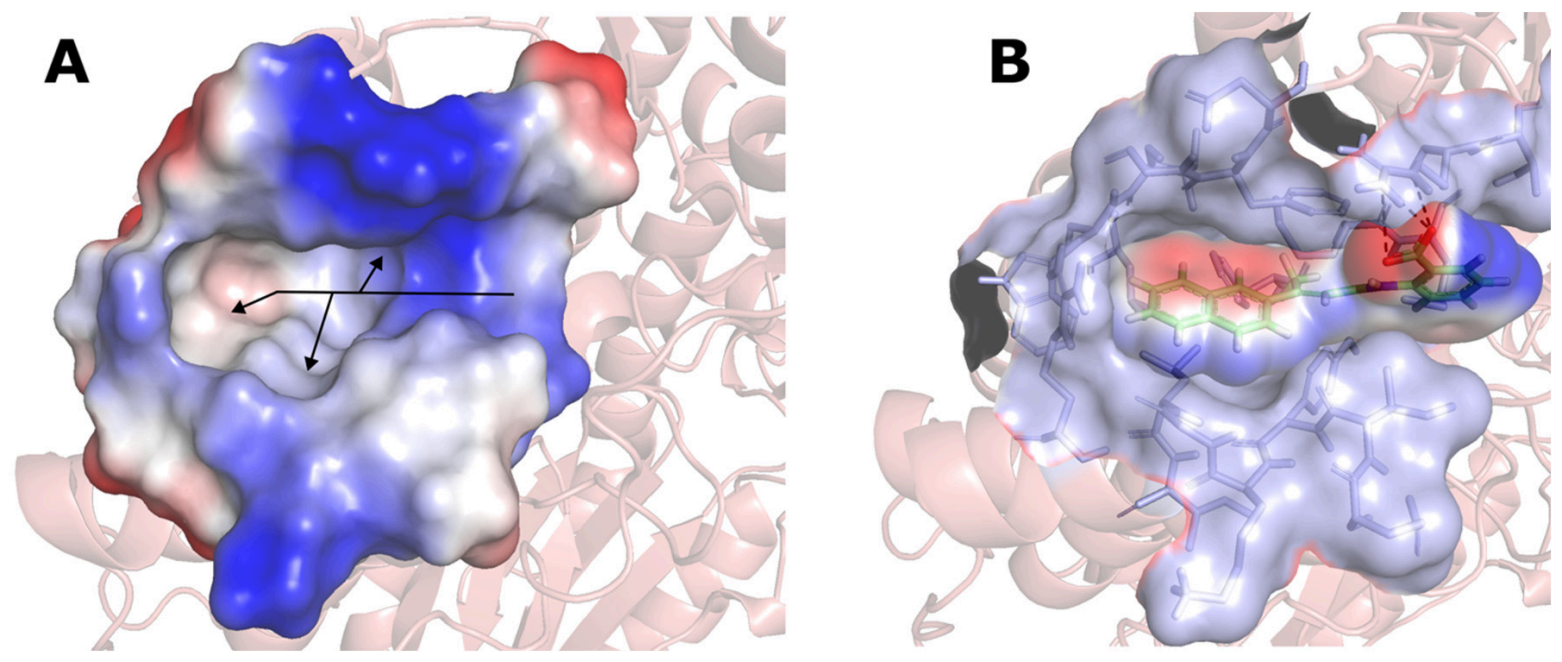
Electrostatic potential map of the telomerase inhibitor binding pocket. (**A**) Surface representation coloured by electrostatic potential (red: negative; blue: positive). Black arrows indicate the left-hand and right-hand sub-pockets discussed in the text. (**B**) Binding mode of BIBR1532 within the tcTERT BIBR1532-binding pocket (light blue transparent surface), overlaid with the electrostatic potential map. Black dashed lines indicate hydrogen bond interactions.

To validate the docking protocol, we performed redocking of the co-crystallised ligand BIBR1532 (PDB 9Q14) using AutoDock Vina, Vinardo, and GNINA. The root-mean-square deviation (RMSD) between the crystallographic pose and the best-scoring docked pose was 0.63 Å (Vina), 0.57 Å (Vinardo), and 0.67 Å (GNINA), confirming that all three methods accurately reproduce the experimental binding mode. Both molecular docking results with electrostatic potential analysis of the tcTERT BIBR1532-binding pocket suggest that the naphthalene moiety may engages the left-hand sub-pocket with high geometric complementarity (Figure 4). A second ring system bearing a carboxyl group, situated on the right-hand side of the binding pocket, serves as the principal mediator of hydrogen bond formation. Deprotonation of this group at physiological pH is particular important, as it facilitates electron delocalisation across the aromatic system. This shifts the electrostatic potential toward more negative values and alters the overall polar character of the compound.

**Figure 5:**
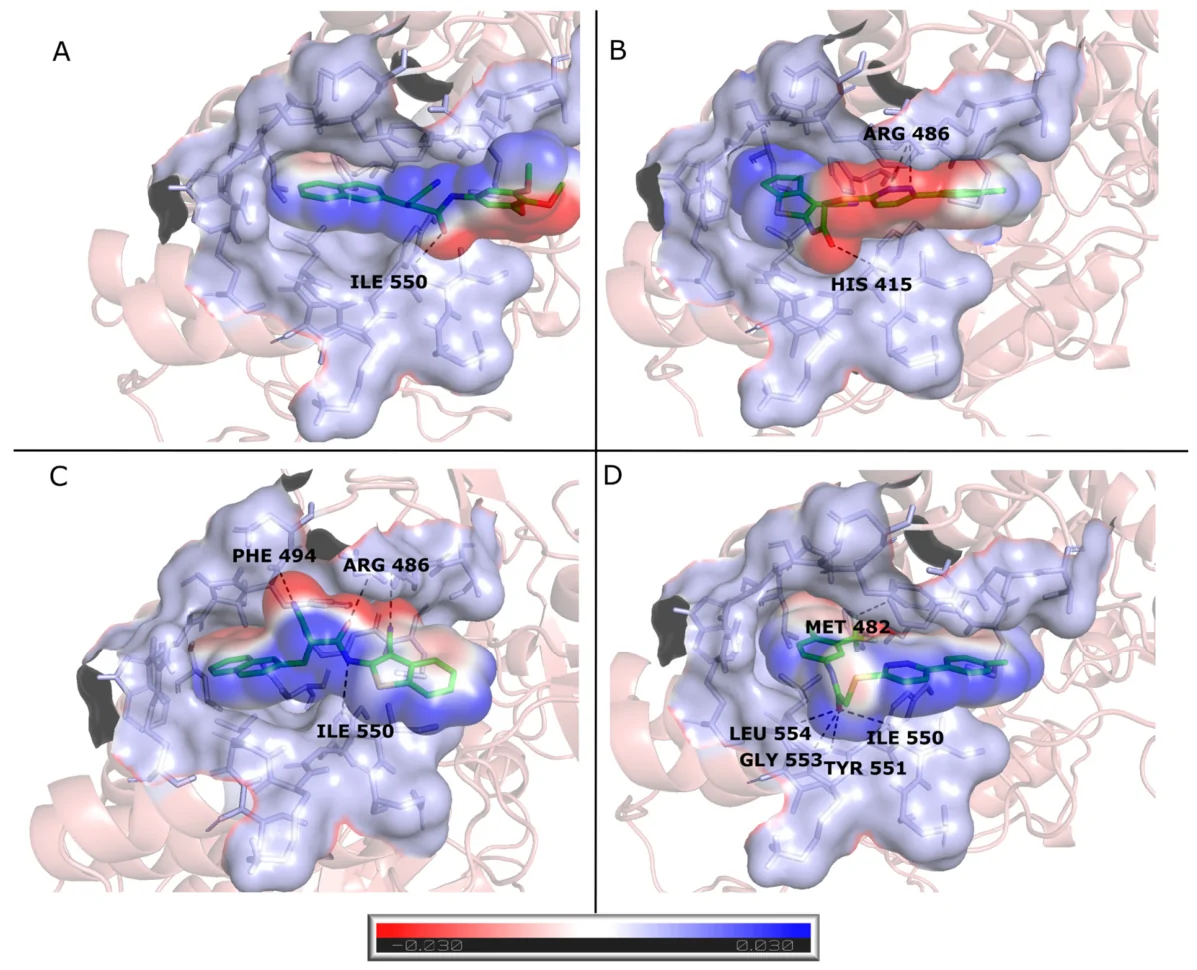
Panels (**A**–**D**) show the binding poses of compounds **39**, **8e**, **36b**, and **4l**, respectively within the same protein pocket. In each panel, the protein binding pocket is rendered as a transparent light blue surface, the ligand is depicted using a liquorice representation, and an electrostatic potential map is superimposed on the ligand (red: negative; blue: positive). Black dashed lines indicate predicted hydrogen bonds between the ligands and the residues labelled on the figure. In each panel, the planar two-membered ring is accommodated within the left-hand subpocket, while the remainder of the molecule extends into the right-hand subpocket, where it engages in potential hydrogen-bonding interactions.

Molecular docking with tcTERT receptor of the four most active compounds identified in the analysed studies was performed, and electrostatic potential maps were calculated to provide further insight into their interactions with the tcTERT BIBR1532-binding pocket. Figure 5 shows that the studied ligands probably engage the deep left-hand sub-pocket through either a fused aromatic ring system or a halogen-substituted benzene ring.

Compounds **8e** and **4l**, in contrast to the remaining ligands, demonstrate high geometric complementarity with the central sub-pocket, forming hydrogen bonds via a carbonyl and a nitrile group, respectively. Except for compound **36b**, all ligands exhibit favourable accommodation within the right-hand sub-pocket through a planar aromatic ring system with partial rotational freedom. The substitution pattern of compound **39** suggests a capacity for additional hydrogen bond formation; however, this was not captured by the docking analysis. A comprehensive evaluation of the hydrogen-bonding potential of the investigated ligands would require sufficiently extended molecular dynamics simulations.

Compound **36b** warrants particular attention. Its energetically more favourable docking poses position the 2-cyanothiophenecyclohexane moiety within the right-hand sub-pocket, while interaction with the left-hand sub-pocket is maintained through the fused aromatic ring system, consistent with the other compounds. Slightly higher energy poses with inverted orientation of the compound **36b** were also identified. This suggests that the ligand may not have a strong directional preference within the binding pocket. The table below provides the information about the binding affinity to the tcTERT BIBR1532-binding pocket (Table 2).

**Figure 6:**
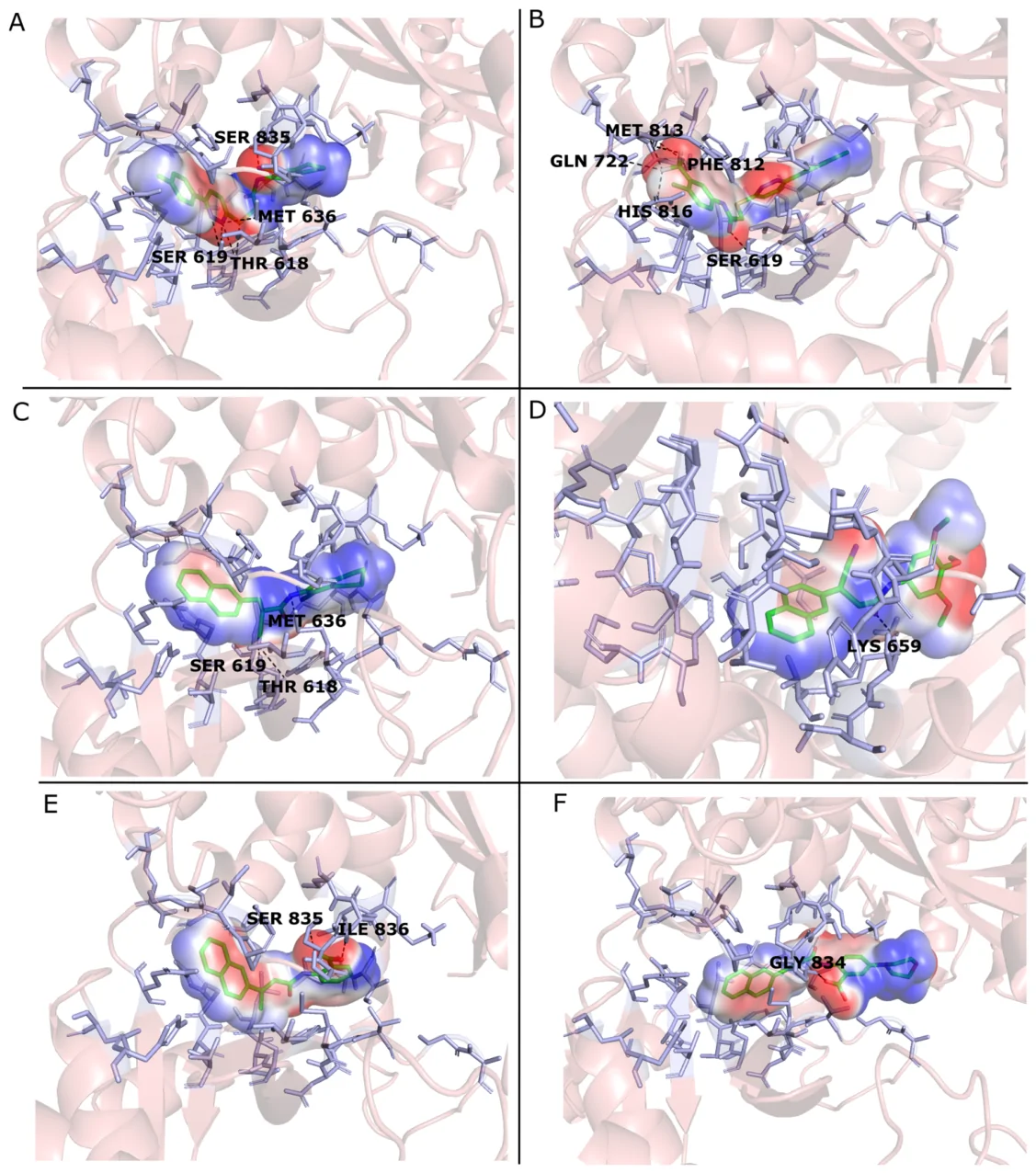
Panels (**A**–**F**) show the binding poses of compounds **8e**, **4l**, **36b**, **39**, BIBR1532 and BIBR1591 respectively, within the human TERT BIBR1532-binding pocket. In each panel, the protein binding pocket and the ligand are rendered as a light-blue stick representation, and an electrostatic potential map is superimposed on the ligand (red: negative; blue: positive). Black dashed lines indicate predicted hydrogen bonds between the ligands and the residues labelled on the figure. In each panel, the planar two-membered ring is accommodated within the left-hand subpocket, while the remainder of the molecule extends into the right-hand subpocket, where it engages in potential hydrogen-bonding interactions.

Recent cryo-EM work by Wang et al. provided important structural insight by showing that BIBR1532 binds to a human TERT BIBR1532-binding pocket distinct from the pocket previously proposed based on the *T. castaneum* telomerase model [26]. Earlier efforts to optimise BIBR1532 derivatives have produced either limited improvements in activity or results that were difficult to interpret. BIBR1591 contains an additional morpholine moiety that could potentially introduce additional hydrogen-bonding interactions and improve steric complementarity within the binding pocket. Nevertheless, it displayed substantially weaker telomerase inhibition than BIBR1532. To understand that phenomenon, we performed docking studies with the use of recently published human model (PDB 9Q14) as receptor. Figure 6 presents the molecular docking results obtained for four selected derivatives **39**, **8e**, **36b**, and **4l**, together with the reference compounds BIBR1532 and BIBR1591.

The structure of BIBR1532 was obtained directly from the Protein Data Bank. The selected BIBR1532 derivatives were generally planar and adopted a characteristic V-shaped conformation within the binding pocket. In contrast, compound **39** did not fit well into the human TERT BIBR1532-binding pocket, presumably because its three methoxy substituents introduced steric clashes. A similar trend was observed for the BIBR1591 derivative: its morpholine ring adopted an orientation approximately perpendicular to the aromatic scaffold, suggesting a possible steric penalty that may contribute to lower telomerase inhibitory activity. Thus, our analysis partially interprets results obtained in vitro. The table below provides the binding affinity of all compounds to the human TERT BIBR1532-binding pocket (Table 3).

## 4. Discussion

In our work, we reviewed previous efforts to identify improved BIBR-related telomerase inhibitors. An ideal derivative should combine high telomerase inhibitory activity with favourable pharmacokinetic properties. Previous structure-guided studies were largely based on two assumptions. First, new derivatives were designed as structural analogues of BIBR1532, which is commonly used as a reference compound. The second assumption was to use the *T. castaneum* TERT receptor for docking as well as molecular modelling studies. A recent study by Wang et al. showed that this assumption was not correct. Despite the high degree of TERT conservation, BIBR1532 binds at a different site in human telomerase. Despite this, many studies over the years have been at least partially successful, as structural analogues of BIBR1532 to some extent directly inhibited human telomerase in *T. castaneum*. Nevertheless, among the compounds discussed here, BIBR1532 remains one of the most potent direct biochemical inhibitors of human telomerase. This does not necessarily translate into superior cellular or in vivo activity, as these effects also depend on the compound permeability, solubility, intracellular exposure, and pharmacokinetic properties. However, it should be emphasised that, according to our docking studies, the mechanism of binding may be completely different. Some derivatives, such as BIBR1591, showed substantially weaker telomerase potency than BIBR1532, which was difficult to explain based on the *T. castaneum* model. The recent discovery of BIBR1532 binding to human telomerase has provided a significant new direction for our research. Our molecular docking results clearly show that some derivatives, despite their structural similarity to BIBR1532, do not exhibit an optimal geometric fit within the recently identified human TERT BIBR1532-binding pocket. For instance, BIBR1591 contains an additional morpholine ring oriented approximately perpendicular to the rest of the molecule. This orientation may hinder effective binding within the human TERT BIBR1532-binding pocket. A similar effect was observed for compound **39**, in which three methoxy substituents prevent effective binding. These considerations are further supported by the predicted binding affinities for the human TERT BIBR1532-binding pocket. BIBR1591 showed only modest predicted binding, with an AutoDock Vina score of −6.14 kcal/mol and an unfavourable positive GNINA score of 1.99. In contrast, BIBR1532 exhibited strongly favourable binding affinity values, consistent with previous literature reports. Compounds **39**, **8e**, and **4l** showed predicted binding affinities in the range of −8 to −11 kcal/mol, indicating binding strengths comparable to that of BIBR1532. Furthermore, improved geometric complementarity within the binding pocket alone may not be sufficient to explain increased telomerase inhibition. Unfortunately, full molecular dynamics analysis was not possible due to the significant incompleteness of the pdb structure of 9Q14. A specific geometry that prevents the initial step of the enzyme-catalysed reaction from occurring is imperative. Future structure–activity relationship studies of BIBR1532 derivatives should be guided by a framework distinct from that employed previously [17,18,19]. Rather than being determined predominantly by the electronic effects of substituents, the inhibitory activity appears to depend primarily on the molecular shape and conformational rigidity. The newly identified spatially restricted binding pocket imposes substantial geometric constraints on candidate ligands. For example, Al-Karmalawy et al. [18] reported that methoxy and bromo substituents decreased telomerase inhibitory activity; however, this observation was difficult to rationalise using the previously adopted binding model. In the context of the newly proposed pocket, the bulky bromine atom may sterically impede ligand entry or proper accommodation within the binding site. Similarly, methoxy substituents may be detrimental because their conformational flexibility can compromise the geometry required for productive binding. The most active derivatives were predominantly planar molecules capable of adopting a V-shaped conformation. Accordingly, future optimisation should prioritise the improvement of pharmacokinetic properties while preserving a planar scaffold, conformational rigidity, and the ability to attain this V-shaped geometry. In contrast to previous strategies emphasising the modulation of substituent electron-donating and electron-withdrawing properties to strengthen intermolecular interactions, future efforts should focus on structural features that enhance inhibition of the initial stage of telomerase catalytic activity. Taken together, our findings offer novel insights into the development of new human telomerase inhibitors.

## 5. Conclusions

In conclusion, our study provides valuable insights into the reasons why some of the BIBR1532 derivatives studied over the past few years have demonstrated limited activity. Molecular docking suggested that telomerase inhibitory activity may depend on favourable geometric complementarity within the narrow elongated human TERT BIBR1532-binding pocket, together with a ligand orientation compatible with interference in the first step of the enzyme-catalysed reaction. These findings may change the current paradigm for the design of human telomerase inhibitors.

## 6. Limitations

It should be noted, however, that although the 9Q14 cryo-EM structure provides important structural evidence for BIBR1532 binding to human telomerase, the deposited model is incomplete, with substantial portions of the experimentally present macromolecular components not represented in the atomic coordinates. Consequently, 9Q14 cannot be used directly as a complete starting structure for molecular dynamics simulations aimed at a rigorous characterisation of protein–ligand interactions. Such simulations would require careful reconstruction and modelling of unresolved regions, as well as appropriate preparation and validation of the complete telomerase–ligand system. The 9Q14 entry contains 1475 modelled residues compared with 2054 deposited residues, supporting this limitation.

## Figures and Tables

**Table 1:** The comparison of methodology and inhibition activity against human enzyme.

Methodology	Compound	Activity	
		IC50	[%]
TRAP-based assay [16]	**36b**	0.3 μM	-
	BIBR1532	0.2 μM	-
Real-time PCR telomerase detection kit [17]	**8e**	-	58.36
	BIBR1532	-	65.45
Quantitative telomerase detection kit [19,20]	**4l**	-	64.95
	BIBR1532	-	72.74
	BIBR1591	-	68.75
TRAP-PCR-ELISA [21]	**39**	0.62 ± 0.12 μM.	-
	BIBR1532	0.32 ± 0.07 μM	-

**Table 2:** Binding affinity of all studied compounds to tcTERT BIBR1532-binding pocket (scoring function: Vina).

Compound	Binding Affinity (kcal/mol)
**39**	−7.5
**8e**	−9.3
**36b**	−8.1
**4l**	−8.1

**Table 3:** Binding affinity of all compounds to the human TERT BIBR1532-binding pocket computed using three different scoring functions: Vina, Vinardo and GNINA.

	Binding Affinity (kcal/mol)		
Compound	Vina	Vinardo	GNINA
**39**	−8.1	−4.98	−8.21
**8e**	−9.3	−6.48	−9.46
**36b**	−10.9	−6.72	−5.8
**4l**	−9.8	−5.75	−8.91
BIBR1532	−11.89	−9.71	−11.90
BIBR1591	−6.14	−6.14	1.99

## Data Availability

All data generated or analysed during this study are available from the corresponding author by request.
